# USING A COMMUNITY ADVISORY BOARD TO DEVELOP A TELEHEALTH CONCIERGE FOR RURAL OLDER CANCER SURVIVORS

**DOI:** 10.1093/geroni/igad104.0753

**Published:** 2023-12-21

**Authors:** Revika Singh, Francesca Rosen, Marquita Lewis-Thames

**Affiliations:** Northwestern University, Chicago, Illinois, United States; Northwestern University Feinberg School of Medicine, Chicago, Illinois, United States; Northwestern Feinberg School of Medicine, Chicago, Illinois, United States

## Abstract

Compared to pre-pandemic telehealth utilization, rural telehealth utilization has increased 1700% for some rural healthcare facilities. Yet, rural older adults remain slower to adopt telehealth, partly due to technical knowledge barriers. To identify practical solutions to overcome these navigation barriers for rural older adults, perspectives about telehealth implementation by older adults are necessary but remain a gap in research. To this end, this project adopts a user-centered design approach to develop a relevant, end-user-informed telehealth navigation tool for rural older cancer survivors with cancer-related distress through a community advisory board (CAB). We identified and selected CAB members from May-September 2022 from community-based organizations (CBOs) serving rural counties and zip codes in northern and central Illinois. CBOs were identified through internet search or snowball recruitment. Operating CBOs and interested community members with rural health interests received an email or telephone call about the CAB to review the roles and responsibilities of the CAB and confirm their interests. To date, over 30 CBOs in rural Illinois were identified, contacted, and mapped according to their rural health resources. We conducted two in-person and three virtual orientations. We identified one mental health professional, one older cancer survivor, one community member, three social workers/patient navigators to join the CAB. Preliminary barriers to telehealth utilization identified through ongoing CAB meetings include inadequate/inaccessible internet infrastructure, variable technical literacy, and concerns about trust-building and confidentiality. Ultimately, community advisory boards can be an effective approach in bridging community and academic partners to improve telehealth navigation for older rural adults.

